# 

**Published:** 1889-11

**Authors:** 


### Society Meetings.

THE MEDICAL SOCIETY OF THE STATE OF NEW YORK.—The next annual meeting will be held in Albany, February 4–6, 1890. The Business Committee has been appointed as follows: Dr. Geo. H. Fox, 18 East Thirty-first street, New York; Dr. Henry Flood, Elmira; Dr. Herman Bendell, Albany.

Applications should be made *before January 1st* by those desiring to secure the proper arrangement of time and subjects. Papers should not exceed fifteen minutes, and the title should accompany the application, which may be made to any member of the committee.

DANIEL LEWIS, *President.*

62 PARK AVENUE, NEW YORK, October 10, 1889.

The Southern Surgical and Gynecological Association will hold its second annual meeting in Nashville, Tenn., November 12, 13, and 14, 1889. This meeting will, no doubt, be a successful one, as upwards of thirty papers, by distinguished authors, are announced.

---

The McDowell Medical Society will hold its annual meeting at Henderson, Ky., November 11 and 12, 1889.

### BOOKS AND PAMPHLETS RECEIVED.

A Text-Book of Human Physiology, including Histology and Microscopical Anatomy; with special reference to the Requirements of Practical Medicine. By Dr. L. Landois and William Stirling, M. D., Sc. D. Third American, translated from the sixth German, edition. Philadelphia: P. Blakiston, Son & Co. 1889.

A Text-Book of Animal Physiology, with introductory chapters on General Biology and a full Treatment of Reproduction. For students of Human and Comparative (Veterinary) Medicine, and of General Biology. By Wesley Mills, M. A., M. D., L. R. C. P. (Eng.), Professor of Physiology in McGill University and the Veterinary College of Montreal. With over 500 illustrations. New York: D. Appleton & Co. 1889.

A Manual of Chemistry for the Use of Medical Students. By Brandreth Symonds, A. M., M. D. Philadelphia: P. Blakiston, Son & Co. 1889.

The Story of the Bacteria and their Relations to Health and Disease. By T. Mitchell Prudden, M. D. New York and London: G. P. Putnam's Sons. 1889.

The Urine, The Common Poisons, and The Milk. Memoranda, Chemical and Microscopical, for laboratory use. By J. W. Holland, M. D. Illustrated. Third edition. Philadelphia: P. Blakiston, Son & Co. 1889.

The Surgeon's Handbook. By Francis M. Caird, M. B., F. R. C. S., and Charles W. Cathcart, M. B., F. R. C. S. Illustrated. Philadelphia: P. Blakiston, Son & Co. 1889.

A Reference Handbook of the Medical Sciences, embracing the Entire Range of Scientific and Practical Medicine and Allied Science. By various writers. Illustrated by chromo-lithographs and fine wood engravings. Edited by Alfred H. Buck, M. D. Volume VIII. New York: William Wood & Co.

Chemistry: General, Medical, and Pharmaceutical, including the Chemistry of the U. S. Pharmacopeia. A Manual on the General Principles of the Science and their Applications in Medicine and Pharmacy. By John Attfield, F. R. S., Professor of Practical Chemistry to the Pharmaceutical Society of Great Britain. Twelfth edition. Philadelphia: Lea Brothers & Co. 1889.

The Value of Creosote in Fifty cases of Disease of the Air Passages. By Wm. P. Watson, A. M., M. D. Jersey City, N. J.

Practical Notes on Urinary Analysis. By William B. Canfield, A. M., M. D. Reprint from the *Maryland Medical Journal.* Baltimore.

Ninth Inaugural Address of Clark Bell, Esq., as President of the Medico-Legal Society. Reprint from *Medico-Legal Journal.*

Monomania. By Clark Bell, Esq. Reprint from the *Medico-Legal Journal.*

The Duties and the Compensation of the Local Health Officer. By Henry B. Baker, M. D., Secretary of the State Board of Health, Lansing, Mich. Reprint from the Report of the Michigan State Board of Health, 1889.

Suicide and Legislation. By Clark Bell, Esq. Reprint from the *Medico-Legal Journal.*

Medico-Legal Society of the City of New York. Constitution and By-Laws.

Urinary Calculus and Lithotomy. By Thos. W. Kay, M. D., Scranton, Pa. Reprint from the *Maryland Medical Journal.*

Résumé of the Experience of Seventeen Years in the Operation of Dilating Urethrotomy. By Fessenden N. Otis, M. D. Reprint from the *New York Medical Record.*

The Purulent Conjunctivitis of Infants and Blindness in New York State. By Lucien Howe, M. D. Reprint from the Transactions of the New York State Medical Society. 1889.

The "Perfected Evacuator." By Fessenden N. Otis, M. D. Reprint from the *New York Medical Journal.*

Observations and Experiences involving Rectal Diseases. By E. F. Hoyt, M. D. Read before the New York Medical Society, 1889.

Cases of Suprapubic Cystotomy. By L. Bolton Bangs, M. D. Reprint from the *New York Medical Journal.*

Studies in Intestinal Surgery. By Wm. B. Van Lennep, A. M., M. D., Philadelphia, Pa. Reprint from the *Hahnemannian Monthly.*

Some of the Relations of Humane Societies to Institutions having the Care of Children. Read at the Annual Meeting of the American Humane Association, 1889. By E. V. Stoddard, M. D.

Medical Department University of Wooster, Announcement for 1890. Cleveland, Ohio.

Twenty-third Annual Announcement and Catalogue of the Winter Course of Instruction of the Medical Department of the University of Vermont. Session of 1889–'90. Burlington.

Thirty-fourth Annual Announcement of the Kentucky School of Medicine, Louisville. Session of 1890.

State Board of Health Bulletin, Nashville, Tenn. September 20, and October 20, 1889.

Monthly Bulletin of the New York State Board of Health. August, 1889.

Report of Deaths and Contagious Diseases for the Months of August and September, 1889. Newport, R. I.

Health in Michigan. September, 1889.

Weekly Abstract of Sanitary Reports. Volume IV., Nos. 39, 40, 41, and 42.

---

### Literary and Other Notes.

SOME time ago the *Detroit Free Press* offered $3,000 in prizes for the three best serial stories sent in before July 1st. The result of this competition has been that Major Joseph Kirkland, of Chicago, Ill., has taken the first prize of $1,600. His story is entitled, "The Captain of Company K." Mr. Kirkland is the author of "Zury, the Meanest Man in Spring County," "The McVeys," and other stories. The second prize goes to Omaha, Neb., and is taken by Mrs. Eliza W. Peattie. Her story is entitled "The Judge." The third prize of $500 was awarded to Elbridge S. Brooks, of Boston, Mass. The title of this story is, "The Son of Issichar."

---

THE TRANSATLANTIC—A MIRROR OF EUROPEAN LIFE AND LETTERS—VOL. I., NO. 1, OCTOBER 15, 1889.—*The Transatlantic* is a new magazine, issued on the 1st and 15th of each month, devoted to European literature. Its aim is to place before the English-speaking public all that is best of European life and letters. The first number contains a fine portrait of Henrik Ibsen, the Norwegian dramatist and poet, and also the first act of a play from his pen.

*The Transatlantic* has only to equal its first number to be one of the first magazines of the land. Published by the Transatlantic Publishing Company, Boston.

---

THE AMERICAN ACADEMY OF MEDICINE is endeavoring to make as complete a list as possible of the Alumni of Literary Colleges, in the United States and Canada, who have received the degree of M. D. All recipients of both degrees, literary and medical, are requested to forward their names, at once, to Dr. R. J. Dunglison, Secretary, 814 North Sixteenth street, Philadelphia, Pa.

---

THE MICROSCOPE, so long and ably edited by Dr. W. P. Manton, and his associates, and published in Detroit, has been removed to Trenton, N. J., from whence it will now issue. Dr. Alfred C. Stokes assumes its editorial management.

